# A Preliminary Appraisal of the Effect of Pumping on Seawater Intrusion and Upconing in a Small Tropical Island Using 2D Resistivity Technique

**DOI:** 10.1155/2014/796425

**Published:** 2014-12-02

**Authors:** Nura Umar Kura, Mohammad Firuz Ramli, Shaharin Ibrahim, Wan Nor Azmin Sulaiman, Muhammad Amar Zaudi, Ahmad Zaharin Aris

**Affiliations:** ^1^Faculty of Environmental Studies, Universiti Putra Malaysia (UPM), 43400 Serdang, Selangor, Malaysia; ^2^Environmental Forensics Research Centre, Faculty of Environmental Studies, Universiti Putra Malaysia (UPM), 43400 Serdang, Selangor, Malaysia

## Abstract

The existing knowledge regarding seawater intrusion and particularly upconing, in which both problems are linked to pumping, entirely relies on theoretical assumptions. Therefore, in this paper, an attempt is made to capture the effects of pumping on seawater intrusion and upconing using 2D resistivity measurement. For this work, two positions, one perpendicular and the other parallel to the sea, were chosen as profile line for resistivity measurement in the coastal area near the pumping wells of Kapas Island, Malaysia. Subsequently, water was pumped out of two pumping wells simultaneously for about five straight hours. Then, immediately after the pumping stopped, resistivity measurements were taken along the two stationed profile lines. This was followed by additional measurements after four and eight hours. The results showed an upconing with low resistivity of about 1–10 Ωm just beneath the pumping well along the first profile line that was taken just after the pumping stopped. The resistivity image also shows an intrusion of saline water (water enriched with diluted salt) from the sea coming towards the pumping well with resistivity values ranging between 10 and 25 Ωm. The subsequent measurements show the recovery of freshwater in the aquifer and how the saline water is gradually diluted or pushed out of the aquifer. Similarly the line parallel to the sea (L2) reveals almost the same result as the first line. However, in the second and third measurements, there were some significant variations which were contrary to the expectation that the freshwater may completely flush out the saline water from the aquifer. These two time series lines show that as the areas with the lowest resistivity (1 Ωm) shrink with time, the low resistivity (10 Ωm) tends to take over almost the entire area implying that the freshwater-saltwater equilibrium zone has already been altered. These results have clearly enhanced our current understanding and add more scientific weight to the theoretical assumptions on the effects of pumping on seawater intrusion and upconing.

## 1. Introduction

Coastal aquifers are fragile in nature, as they constitute a freshwater source which usually lies on top of seawater with a transition zone in between the two water types [1]. These aquifers are generally in direct contact with seawater, which create an equilibrium state between the freshwater and saltwater, making them complex and fragile in nature and also very vulnerable to seawater intrusion. Therefore, poor management of coastal aquifers can easily lead to the distortion of the equilibrium state that lies between two water types and will eventually hinder economic development and destroy the ecosystem of the affected areas [2]. Moreover, such types of aquifers often contain limited groundwater resources, making their management a very critical task that necessitates special consideration to curtail or prevent saltwater intrusion into the aquifer or encroachment of saltwater towards pumping stations as a result of upconing [1]. Here, the term “saltwater upconing” refers to the upward movement of deep saltwater into freshwater aquifers which result from pressure changes in response to pumping. There had been many reports of similar incidents at different coastal areas all over the world whereby a decrease in groundwater quality was linked to groundwater pumping [3].

Human activities, such as groundwater extractions, are believed to be responsible for lowering the groundwater level and subsequently altering the natural balance that lies between freshwater and saltwater in coastal aquifers, thereby reducing the flow of fresh groundwater to coastal waters, and eventually leading to saltwater intrusion [4]. Among all the human activities, pumping has been identified as the major cause of saltwater intrusion and upconing in coastal areas [4–6]. Other than upconing, an upward movement of saltwater can also take place due to dispersion. A continuous pumping of groundwater indiscriminately for a long period may result in saltwater encroachment into the pumping well [7]. The effects of seawater intrusion and upconing can persist even after the groundwater pumping has long been stopped, since it takes a longer period of time for an aquifer to recover to its initial state. As a consequence, the clay matrix of the affected area may be altered and thereby distort the aquifer, which represents a change which is too expensive to repair [8].

The development of a plan for the best possible usage of groundwater in the coastal area for water supply necessitates a preliminary assessment of the factors controlling seawater intrusion in the coastal areas, depending on the variation of the elements of water balance which includes pumps for fresh- or seawater into or out of the aquifer. Changes in the speed and volume of pumping in or out of the aquifer are associated with a number of temporary deformation processes that can spread along the interface that lies between fresh- and seawater [5]. These require an in-depth understanding of the whole processes and factors influencing coastal hydrogeology. However, the contemporary knowledge and perception of saltwater upconing are primarily derived from theoretical assumptions which mostly involve laboratory experiments such as reconstructions of aquifer and how flow reacts to groundwater pumping, in addition to a few numbers of actual field-measurement and laboratory analysis. This is perhaps due to the difficulties and complexity associated with field-based studies of salt transport dynamics taking place underneath pumping wells [9, 10]. Hence, one of the problems that may arise with respect to laboratory experiments is that aquifers may not be as homogeneous as most of the lab materials. Moreover the laboratory experiments are in a controlled environment; therefore many important factors that can influence the processes of upconing and seawater intrusion might be unaccounted for. Hence, there is a need for direct examination of saltwater upconing, considering the substantial and intrinsic uncertainties connected with this phenomenon and diffused behaviour of solute transport [9].

On the one hand, it might be right to say that numerical models like MODFLOW will provide more practical explanation of the groundwater flow and contaminants transport [11]. The MODFLOW which was originally developed by the U.S. Geological Survey (USGS) has been used by many researchers worldwide in an attempt to make sense of what exactly is happening beneath the ground with respect to seawater intrusion [12]. For example, a three-dimensional numerical model for flow and solute transport was used to create a simulation model which was then employed to predict the spatial distribution of salinity and mass balance based on different scenarios for the period of 2006–2020 [11]. MODFLOW was also employed by [13] to assess the seawater intrusion in the coastal aquifer of Ravenna (Italy) while [14] uses the MODFLOW SEAWAT code to evaluate the extent of seawater intrusion in the Gulf coast aquifers of Alabama in USA.

However, on the other hand, MODFLOW and other numerical models not only are complex in nature, but also require a large amount of data which more often than not is rarely available. This problem is not limited to areas with little or no data but the customary complaint of insufficient data will persist even in areas with large amounts of data [4]. Finding data to feed the requirements needed to execute such models is mostly unrealistic in areas like Kapas Island where little information is available regarding the groundwater system of such areas [15, 16]. Lack of sufficient data in hydrological modelling can hinder a model's ability to predict and produce reliable results; consequently, it adds to the uncertainties that may arise as a consequence of a lack of calibration and validation [17]. Thus, there is a need to employ other methods to address the issues which arise from groundwater movement and contaminant flow, because the analytical solutions are rarely achievable except in areas with very simple systems [18].

On the one hand, the electrical response of a variety of earth materials near the ground surface can be measured through resistivity surveys, enabling researchers to determine the hydrogeological structures and map out subsurface geological layers. On the other hand, seawater is characterized by a high concentration of sodium chloride, which is known to have electrolytes, and these electrolytes make the resistivity of seawater very low. Thus, electrical resistivity technique is considered to be a reliable method in detecting and delineating seawater affected areas [19]. Many researchers have also used electrical resistivity techniques to assess seawater intrusion [8, 20–23]. However, to the best of the author's knowledge, there is only a limited number of studies which involved the use of 2D resistivity method “if any” to capture upconing or seawater intrusion that resulted from pumping and aquifer freshwater recovery during or after pumping. Therefore, this work aimed at using 2D resistivity measurement to capture the upconing and seawater as it intrudes into the aquifer just after pumping and then the processes of freshwater recovery in the aquifer.

## 2. Study Area

**Figure 1:**
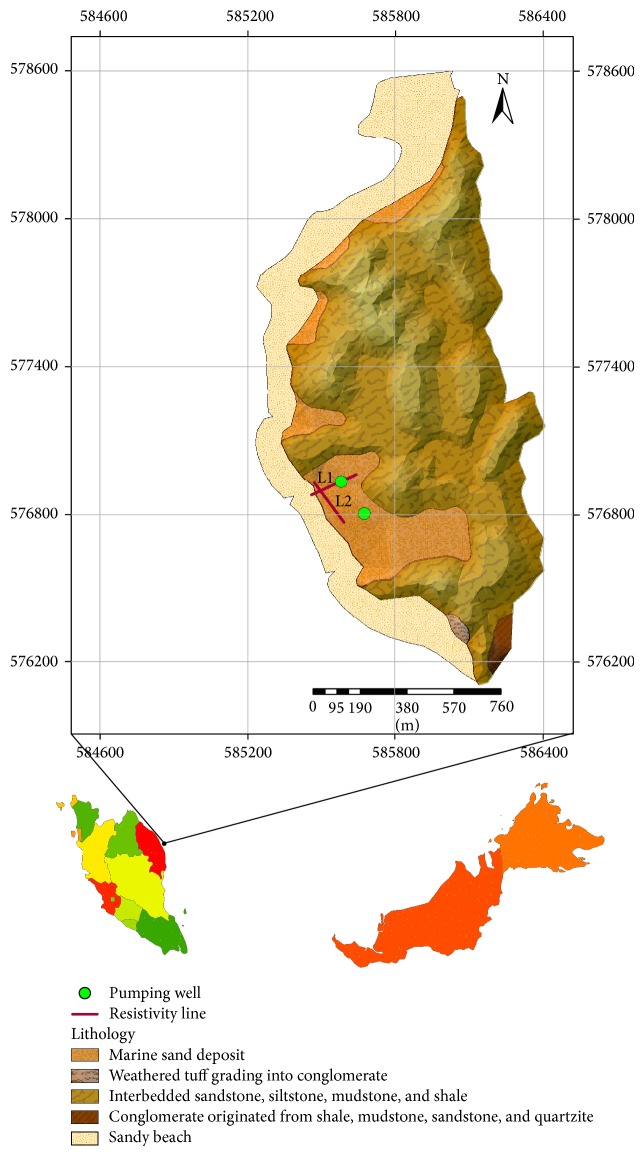
Location of Kapas Island in Malaysia and map showing its elevation, pumping wells, and resistivity lines.

Kapas Island (Figure 1) was estimated to be 2 km^2^, mostly hilly up to an altitude of 100 meters (m). This island is located at 5°13.140′N and 103°15.894′E and under the jurisdiction of Terengganu State, in the north-eastern part of Malaysia. The island receives over 2,800 mm of rainfall per annum, mostly during the monsoon season between November and February. The island's humidity can reach up to 80% while the temperature fluctuates between 28 and 31°C.

The island (Figure 1) mostly consists of sedimentary rock that is graded into low grade metamorphic rock equivalent to metasediment and can generally be classified into two units based on age: conglomerate and interbedded layers of shale, siltstone, sandstone, and mudstone. The latter parts are the dominant rock form on the island and they predate the conglomerate. In contrast, in the southern part of the island, conglomerate appears in a small segment. The clastic components of the conglomerate were produced by combinations of shale, quartzite, mudstone, and sandstone. In addition, tectonic movement had created the succession pattern of layers that creates tight folds with their axes crossing north (N) to north-north west (NNW).

At the western part of the island, alluvial and marine sand lie within the beach site. Layers of fine and medium-sized sand with coral, clay, and shell combine to form semiconsolidated alluvia. The geological logs [24] taken from the alluvial deposits reveal that only one aquifer (the unconfined aquifer type) exists in the area, though it is formed by layers of different materials such as sand, coral, and shell. The logs also clearly show that the northern part of the deposits is the sandiest part of the island mixed with coral and shell. The top layer covers a depth of up to 2 m and mostly consists of loamy sand. Below this lies a layer of semiconsolidated sand formed by a mixture of cemented shell and coral up to 4 m depth and the aquifer starts from this layer. The next layer comprises varying sizes of shell and coral with changeable compositions of sands and clays alongside these layers [2].

## 3. Materials and Methods

### 3.1. Field Survey for Resistivity Measurement

The field survey for electrical resistivity was carried out in two phases, with phase one taking place in June 2012 during a reconnaissance survey where only one line, 160 m long, was measured using an ABEM Terrameter SAS 1000/4000 (microprocessor-driven resistivity meter). The result of this first fact-finding measurement led to the second phase survey but, this time, a longer cable was used for the profile lines (200 m). This is because the result of the first survey showed that the cable did not reach close enough to the seashore and, as such, it could not capture the intrusion of seawater into the aquifer in detail. In both surveys, the configuration Wenner array was employed due to its strong signal strength and its sensitivity in distinguishing vertical changes and horizontal structures [19, 25] and its ability to delineate areas affected by seawater intrusion [26].

The second phase of electrical resistivity measurement was conducted in March 2013 during the postmonsoon season. It should be mentioned that this timing was purposely planned to correspond with less human activity and more recharge to the island's aquifer. This is to ensure that any remnants of past saltwater intrusion have been flushed out of the aquifer so that it will not influence the current result. In so doing, the effect of pumping on seawater intrusion and upconing would be captured as it happens, in addition to getting a clear view of the process of recovery and how the freshwater pushes back seawater out of the island's aquifer. At the beginning of this experiment, 2 subpermanent profile lines were positioned; one was set perpendicular to the sea (inland ward across the pumping well), while the second line was set parallel to the sea about 50 m away from the seashore. Each line was set with two cables of 100 m each to form a straight 200 m long line; then electrodes (made from stainless steel) were pinned to the ground at a fixed distance of five meters along each of the two profile lines. Next, the cables and the electrodes were connected with jumpers. The cables were then connected to the resistivity meter at the centre of the line. From these two positions (L1 and L2), six resistivity measurements (three each) were carried out at four-hour intervals (time-series measurements). It is worth mentioning that prepumping measurements were not feasible in this particular situation. This is due to the fact that the pumping machine automatically starts pumping once the water level at the reservoir reaches a certain level. Moreover, the groundwater pumping is usually conducted two to three times a day.

Firstly, groundwater was continuously pumped at a rate of 45 m^3^ d^−1^ for about 5 hours (from 7:00 am to 12:00 pm), and then the first measurement was taken immediately after the pumping was switched off. Then the resistivity meter was quickly transferred to the second line (L2) parallel to the sea and, subsequently, the resistivity of that profile line was also measured. This procedure was repeated four hours later for the second measurement at each of the two lines (L1 and L2). The final measurements from these two lines were conducted after another four hours. The measured data was later imported into the RES2DINV software and subsequently a least-squares inversion technique was used to produce the 2D resistivity images. This inversion technique was chosen due to its ability to provide the best possible result in areas such as seawater intrusion or a transmission edge of a chemical plume which are usually associated with a smooth variation [19].

## 4. Results and Discussion

### 4.1. Resistivity Profiles

**Figure 2:**
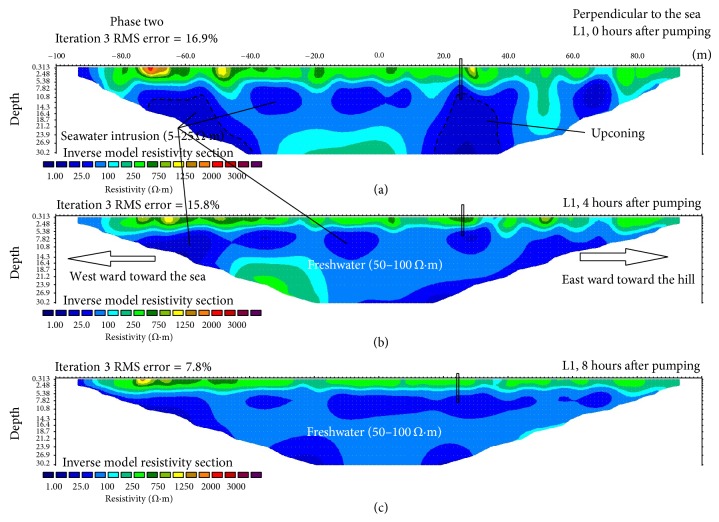
Electric resistivity profiles phase 2 for line L1 perpendicular to the sea at 0 hours (a); 4 hours (b); and 8 hours (c) after pumping, respectively.

The first resistivity line in the phase two measurement (0 hours after pumping) also shows a somewhat similar result to that of the phase one resistivity line (Figure 2(a)). The top layers down to a depth of 3 m represent the dry sand with resistivity ranging from 200 to 3000 Ωm but were mostly dominated by 250 Ωm. The next layer constitutes fine sand, coral, and shell [24] representing a partially saturated zone with a resistivity of 150 Ωm. This is followed by the main aquifer between 7 and 30 m depth with a resistivity value of 50 Ωm representing freshwater [27] and, just like the one above it, this layer also comprises sand, coral, and shell. Within this layer from the left, there is an inward feature with a low resistivity of 10 Ωm indicating seawater intrusion. Then, 20 m away from this feature, there are two separate features with the same resistivity values of 25 Ωm indicating the dilution of saltwater. The two features also give the impression that they originate from the sea and move towards the pumping well, suggesting that the intrusion might have been influenced by pumping [5] but as the saline water moves toward the pumping well, it tends to be diluted before it reaches the pumping well [7].

A very good feature of an upconing can clearly be seen at about 125 m on the line just below the pumping well with varying layers of resistivity indicating degrees of salinity at the area [9]. This salinity zone featured a reversed “u shape” figure which begins at 6 m depth with a low resistivity value of 1–10 Ωm. It is worth mentioning that the lithology of this area does not contain clay materials; as such, the low resistivity zone beneath the pumping well can only mean one thing: “upconing” [7, 9]. Thus it is beyond coincidence that the lower resistivity in this area appeared after a continuous groundwater pumping for more than 5 hours. The outermost layer of the upconing feature shows a resistivity of 25 Ωm signifying dilution of saltwater. Then, this is followed by another layer of lower resistivity value (10 Ωm) indicating an increase in the salinity of water at that area. Beneath this layer lies the lowest resistivity layer (1 Ωm) indicating a high concentration of salinity [21, 27].

The second measurement of this profile line was conducted 4 hours after the first measurement is shown in Figure 2(b). This resistivity profile line shows that the concentration of saline water has significantly decreased and the resistivity line is now dominated by freshwater 50 Ωm [22]. First, the feature that represents the upconing has almost disappeared with only a little left at the bottom of the resistivity image. Additionally, the brackish water is now moving towards the sea as indicated by the three separated zones of 25 Ωm. This implies that the aquifer is recovering and therefore the freshwater is flushing out the saline water. However, at the left side of the resistivity image there is still some portion of saltwater intrusion (10 Ωm) that remains.

The last measurement on this line was taken eight hours after the groundwater extraction discontinued (Figure 2(c)). Like its predecessor, this resistivity image is also dominated by freshwater (50 Ωm). At this time the freshwater has successfully flushed out most of the high concentrated saline water out of the aquifer. However, the saline water intrusion has left some traces of salinity, as somewhat a straight line appears showing low resistivity in the area (25 Ωm). This shows that the effects of seawater intrusion persist to some extent even after the pumping ended and may take some time before the aquifer regains its original status [8]. It is noticeable that the water level tends to increase with time as shown in the resistivity images of this profile line with the 0 hours (Figure 2(a)) being the lowest seven m depth, which is then elevated to around 5.2 m depth in the second measurement and finally 3.7 m at the last measurement signifying the aquifer's speed of recovery (Figures 2(b) and 2(c)), respectively.

**Figure 3:**
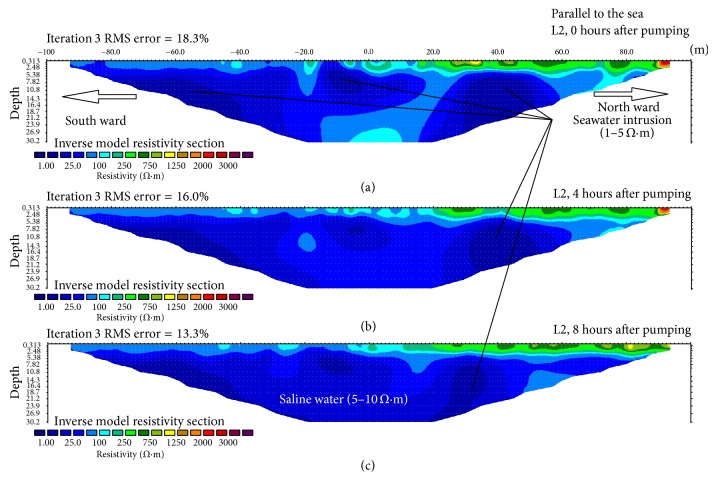
Electric resistivity profiles phase 2 for line L2 parallel to the sea at 0 hours (a); 4 hours (b); and 8 hours (c) after pumping, respectively.

In order to understand how pumping affects the transition zone or freshwater-saltwater equilibrium zone, line 2 (L2) was measured parallel to the sea. The first resistivity image on this line (Figure 3(a)) shows a higher effect of saltwater intrusion compared to the other line with varying low resistivity values covering most of the resistivity line. From the left, a very low resistivity zone (1 Ωm) can be seen. This part corresponds to the second pumping well situated at a parallel distance inland ward as shown in Figure 1, suggesting that the intrusion of seawater at this point is likely to be aggravated by pumping. This layer was sandwiched by another layer of diluted saline water with a resistivity value of 10 Ωm [28]. The case is similar at the centre showing a somewhat spherical shaped feature with 4 layers of different resistivity values. The inmost layer had the least resistivity (1 Ωm), followed by 10, 25, and 50 Ωm. The 2D image also shows a large zone of low resistivity (1 Ωm) at the right hand side. This is the area where line L2 which is parallel to the sea crossed line L1 (perpendicular to the sea) and, due to this crossing, the effect of seawater intrusion is better captured in line L2 compared to profile line L1.

The second and the third measurements on this line took place at about 4 and 8 hours after the first measurement (Figures 3(b) and 3(c)), respectively. The resistivity images show some similar patterns to the first measurement but there are significant variations of the spatial distribution of the resistivity values in relation to time. The resistivity images show that the size of the areas with the lowest resistivity values (1 Ωm) decreases more with time, whereas the 10 Ωm resistivity zone increases. This perhaps is due to the aquifer's recovery which resulted in freshwater flushing out the saline water from the aquifer but when it came to this zone which is likely to be the saltwater-freshwater interface, the pressure from the sea increased [29]. Therefore, instead of the saline water being pushed out of the aquifer, it ends up being diluted by the freshwater pushing from the other end and subsequently alters the equilibrium zone that lies between saltwater and freshwater [30]. As the water reached closer to the sea, the freshwater was not able to flush the saline water out completely but rather it diluted the saline water, which, over time, will increase inland ward and pollutes the aquifer [1, 30].

### 4.2. Validation

**Figure 4:**
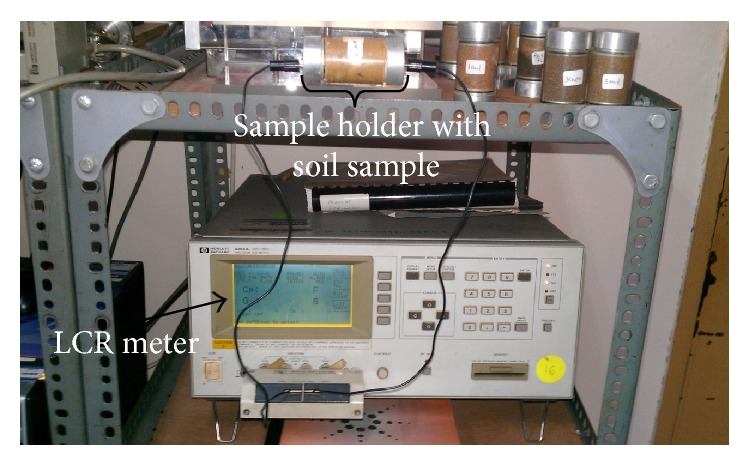
Laboratory measurement for resistivity of soils saturated with groundwater and seawater using a LCR meter.

When conducting environmental assessment, it is always important to test the validity of a model or result because using an unvalidated result may lead to bias and, as a consequence, will cause a wrong management plan [31, 32]. Thus, 12 core soil samples were collected from the area, and, afterwards, an attempt was made to emulate the soil composition of the aquifer in a laboratory based on the lithological information of the area [24]. The soil samples were initially placed into sample holders of different diameters and length and then divided into four groups (3, 3, 4, and 2 samples). The first group consisting of three samples was saturated with seawater collected from the island. The samples of the second group were saturated with the mixture of fresh- and seawater (1 : 1 ratio) collected from the study area. The four samples of the third group were saturated with the island's freshwater while the last two soil samples were still in their natural state and were not saturated with either fresh- or seawater. Afterwards, one after another, the conductance of each of the samples was measured with inductance-capacitance-resistance (LCR) (Figure 4) [33, 34]. The resulting outputs were then converted into resistance via

(1) $$\begin{array}{c} R=\frac{1}{G}, \end{array}$$

where *R* is the resistance and *G* represents the conductance in Siemens (S). The sizes and shape of the samples are believed to have an influence on the values *R* and *G* of each sample. Therefore, the resistivity values were calculated using

(2) $$\begin{array}{c} \rho =\frac{Ra}{d}, \end{array}$$

where *ρ* stands for the resistivity (Ωm), *R* is the resistance (Ω), *a* is the cross-sectional area of the sample (m^2^), and *d* represents the length of the sample (m) [33].

It should be mentioned that the LCR meter measures samples at varying frequencies starting from 20 Hz to 1 megahertz (MHz). However, only the data at 20 Hz frequency was considered for the reason that the 20 Hz frequency was the closest to the 16 Hz frequency that was used with the resistivity meter in the field to measure the profile lines [33].

**Figure 5:**
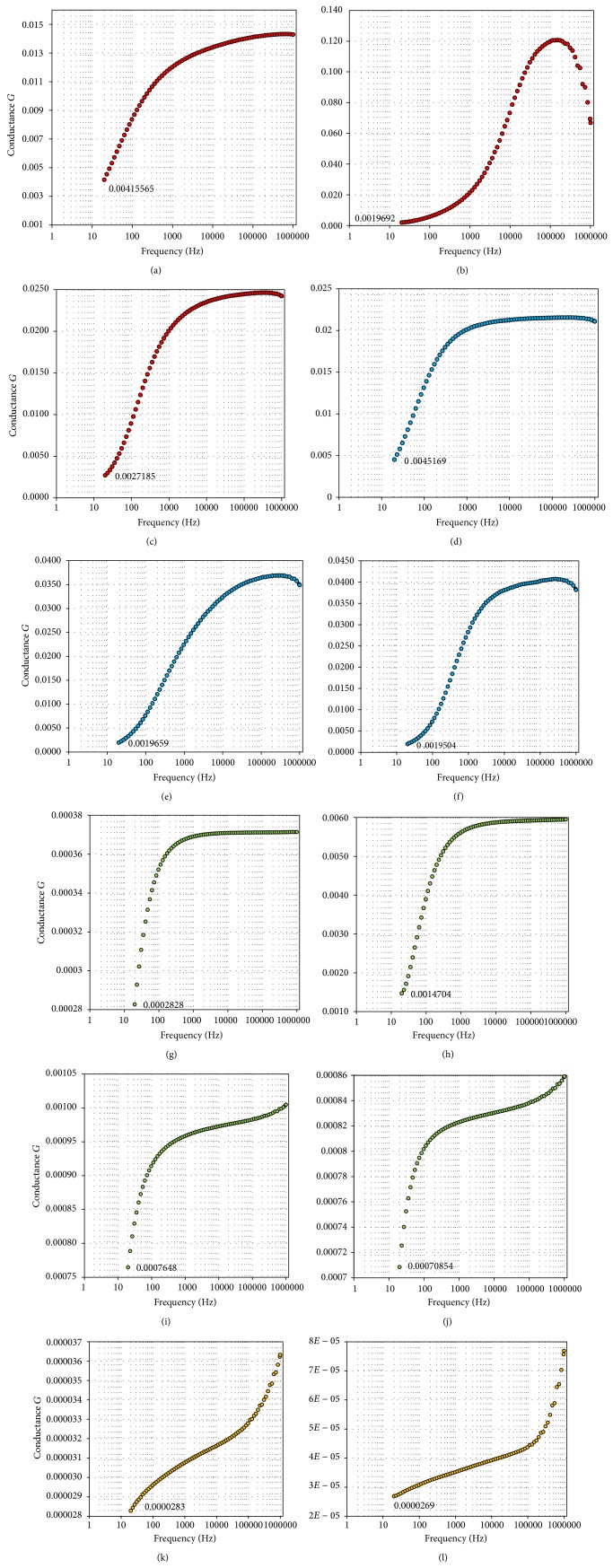
Conductance/frequency graphs from LCR meter measurements ((a)–(c) seawater; (d)–(f) mixed; (g)–(j) freshwater; (k)-(l) soil at natural state).

Table 1 presented the results of the laboratory experimentation which aimed to verify the validity of the field survey and the interpretations of the resistivity images. The results reveal that the resistivity values of the island soils saturated with seawater range between 3 and 6 Ωm (Figures 5(a)–5(c)). These values agree with the interpretation of the seawater and upconing zones resistivity images. Moreover these values are within the known seawater resistivity values [21, 26]. Soils samples saturated with mixture of seawater and freshwater were found to show varying degrees of resistivity ranging between 11 and 27 Ωm (Figures 5(d)–5(f)). These values agree with the earlier interpretation of the resistivity images (Figures 2 and 3) representing dilution or mixture zone [22, 35]. In contrast, soils saturated with the island's groundwater were found to range between 35 and 74 Ωm (Figures 5(g)–5(j)) which is within the agreeable values of freshwater saturated zone [36]. The resistivity values of the island soil at its natural state range between 576 and 1939 Ωm (Figures 5(k) and 5(l)) indicating sand with low content [37]. These findings have further strengthened the interpretation of the resistivity images.

## 5. Conclusion

This work attempted to assess the effects of pumping on seawater intrusion and upconing using 2D resistivity measurements. First, a fact-finding measurement was taken along with a prominent pumping well perpendicular to the sea. The results clearly showed an upconing feature of reversed U. Then, to further understand and strengthen the phase measurement, another field survey was scheduled and two profile lines were set, one perpendicular and the other parallel to the sea, and subsequently six time-series measurements (three each at four-hour interval) were conducted along the two profile lines with the first taken immediately after five hours of pumping.

The results of the first profile line of the phase two measurement showed an intrusion from the sea moving towards the pumping well with low resistivity values in the range of 10 to 25 Ωm. The resistivity image also showed a very low resistivity 1–10 Ωm just beneath the pumping well reflecting an upconing that was believed to be triggered by pumping. As for the recovery and how freshwater pushback resulted in the moving of saline water towards the seashore, the second and the third measurements along this profile line show a gradual decline and the disappearance of the low resistivity areas. Likewise, the L2 profile line situated parallel to the sea along the seashore discloses a very low resistivity with 1–10 Ωm values at the right and left sides of the resistivity image as well as the centre suggesting an active seawater intrusion. Conversely, the second and third measurements along this line reveal interesting information with respect to the processes of recovery and seawater-freshwater interfacing. The results show that as the areas with the lowest resistivity 1 Ωm get smaller with time, the low resistivity (10 to 25 Ωm) tends to engulf almost the entire area meaning that the mixed zone with both freshwater and saltwater is altered by dilution. This could perhaps result from the fact that the freshwater is pushing the saline water out of the aquifer. At the same time, the sea is also pushing back through its pressure which is more intense closer to the shore. As such, the saline water was somehow trapped between two forces which resulted in dilution of the saline water rather than being completely flushed out.

In order to determine the reliability of these findings particularly with respect to the interpretations of the resistivity images, soil samples were placed in sample holders, with some comprising saturated seawater, while others were saturated with either fresh or mixed water from the island and underwent laboratory experiment to measure their respective resistivity values using a LCR meter. The results show a good correspondence between the interpreted images measured in the field and the saturated soils measured in the laboratory. However, it should be stated that the current study lacks the ability to predict future effects that might result from pumping. Secondly, it should be suggested that future research should focus on using multiple lines crossing over one another in order to have 3D images of the upconing seawater intrusion as well as enable researchers to quantify the volume of such intrusion. Nevertheless these results have evidently proven the effects of pumping on seawater intrusion and upconing and improved our present knowledge by adding more scientific value to the theoretical assumptions on the effects of pumping on seawater intrusion and upconing. More importantly, these results show how these processes affect the state of equilibrium that lies between fresh- and saltwater lenses.

## Figures and Tables

**Table 1:** Resistivity values of soil saturated with freshwater and seawater.

Number	Water type	Electrode radius (m)	Length (m)	Resistance	Resistivity (Ωm)
a	Seawater	0.0165	0.053	240.64	3.9
b	Seawater	0.0165	0.087	507.81	5.0
c	Seawater	0.0165	0.053	367.85	5.9
d	Mixed	0.0255	0.039	221.39	11.6
e	Mixed	0.0255	0.039	508.67	26.6
f	Mixed	0.0255	0.039	512.72	26.8
g	Freshwater	0.0165	0.087	3536.58	34.8
h	Freshwater	0.0255	0.039	680.07	35.6
i	Freshwater	0.0255	0.039	1307.56	68.5
j	Freshwater	0.0255	0.039	1411.35	73.9
k	Natural soil	0.0255	0.039	37037.04	1939.0
l	Natural soil	0.0165	0.053	35714.29	576.1
